# Transcriptomics Reveal Antiviral Gene Induction in the Egyptian Rousette Bat Is Antagonized In Vitro by Marburg Virus Infection

**DOI:** 10.3390/v10110607

**Published:** 2018-11-02

**Authors:** Catherine E. Arnold, Jonathan C. Guito, Louis A. Altamura, Sean P. Lovett, Elyse R. Nagle, Gustavo F. Palacios, Mariano Sanchez-Lockhart, Jonathan S. Towner

**Affiliations:** 1 Center for Genome Sciences, US Army Medical Research Institute of Infectious Diseases, Fort Detrick, MD 21702, USA; catherine.e.arnold13.ctr@mail.mil (C.E.A.); sean.p.lovett2.ctr@mail.mil (S.P.L.); elyse.n.abbott.ctr@mail.mil (E.R.N.); 2 Viral Special Pathogens Branch, Centers for Disease Control and Prevention, Atlanta, GA 30329, USA; ypw1@cdc.gov; 3 Diagnostic Systems Division, US Army Medical Research Institute of Infectious Diseases, Fort Detrick, MD 21702, USA; louis.a.altamura2.ctr@mail.mil; 4 Department of Pathology and Microbiology, University of Nebraska Medical Center, Omaha, NE 68198, USA

**Keywords:** transcriptomics, Egyptian rousette bat, Marburg virus, VP35

## Abstract

The Egyptian rousette bat (ERB) is the only known Marburg virus (MARV) reservoir host. ERBs develop a productive MARV infection with low viremia and shedding but no overt disease, suggesting this virus is efficiently controlled by ERB antiviral responses. This dynamic would contrast with humans, where MARV-mediated interferon (IFN) antagonism early in infection is thought to contribute to the severe, often fatal disease. The newly-annotated ERB genome and transcriptome have now enabled us to use a custom-designed NanoString nCounter ERB CodeSet in conjunction with RNA-seq to investigate responses in a MARV-infected ERB cell line. Both transcriptomic platforms correlated well and showed that MARV inhibited the antiviral program in ERB cells, while an IFN antagonism-impaired MARV was less efficient at suppressing the response gene induction, phenotypes previously reported for primate cells. Interestingly, and despite the expansion of IFN loci in the ERB genome, neither MARV showed specific induction of almost any *IFN* gene. However, we detected an upregulation of putative, unannotated ERB antiviral paralogs, as well as an elevated basal expression in uninfected ERB cells of key antiviral genes.

## 1. Introduction

Bats are the second-largest order of mammals on Earth and inhabit diverse biotopes worldwide. In recent years, many bat species have been recognized as reservoir hosts for a wide array of viruses [1,2]. Contact between infected bats and other animals occasionally produces spillover zoonoses that can be highly pathogenic. Among the most notorious emerging pathogens linked to bats are filoviruses, including Marburg virus (MARV) and Ebola virus (EBOV) [3]. Both viruses cause severe diseases in humans and non-human primates (NHPs) with high case fatality rates [4]. Recently, ecological and laboratory efforts have identified the Egyptian rousette bat (*Rousettus aegyptiacus*, ERB) as a natural reservoir for Marburg viruses [5,6,7]. As expected for a viral reservoir, both natural and experimental MARV infection of ERBs cause measurable, low-level viremia and viral shedding but without overt symptoms [8,9,10,11]. In contrast, lethal filoviral diseases in primates are thought to be driven by a strong virus antagonism of host innate immune responses early in infection, particularly interferon (IFN) responses, impairing the host entry into an effective antiviral state to control viral replication [12,13]. This antagonism is controlled by the activities of the EBOV and MARV protein VP35, in concert with either EBOV VP24 or MARV VP40 [14,15,16]. Protein-based studies have suggested that MARV VP35 is overall less inhibitory than EBOV VP35 [14,17], while the MARV infection in vitro suppresses the immune response gene expression to varying degrees dependent on the strain, cell type, and assay [17,18]. However, analogous, antagonism-specific lesions in VP35 of both recombinant EBOV and MARV have been demonstrated to strongly ablate the blockade of immune response and viral growth in vitro, with the VP35-mutant EBOV also showing decreased pathological response in vivo [19]. The similar lack of virulence in ERBs infected naturally with wild-type MARV begets the hypothesis that these animals co-evolved with the virus and have antiviral mechanisms within their innate immune response that temper MARV replication, subsequently preventing the severe disease. Consistent with this hypothesis, the *IFNA* genes were reported to be constitutively expressed in the Australian black flying fox (*Pteropus alecto*), an IFN state proposed to explain how this and possibly other bat species might coexist with resident viruses asymptomatically [20]. Unfortunately, studies of ERB immunology and host-virus interactions in vitro or in vivo have been limited due to a paucity of ERB-specific reagents. However, in recent years, efforts to expand this toolkit have led to the establishment of immortalized ERB-derived cell lines [21], experimental bat colonies and an annotated ERB genome and transcriptome [22,23]. These advances now pave the way for a more rigorous investigation of ERB antiviral systems.

Two studies [17,18] recently incorporated RNA-seq to analyze such antiviral systems in filovirus-infected ERB cell lines. However, they reported striking differences. One [17] showed that MARV (and EBOV) induced significant ERB cell antiviral responses, but a variable, lesser response in human cells. In contrast, the other report [18] found little antiviral gene induction in filovirus-infected ERB cells. While informative and useful, these previous studies used either EBOV, for which ERBs are not a natural host, or a human MARV isolate, which may have adaptations for optimal growth in humans. To address the effect that the MARV infection has on antiviral gene expression in natural host cells, we infected an ERB-derived cell line with a recombinant MARV derived from a wild-caught ERB isolate [24]. In parallel, we investigated a recombinant mutant MARV (MARV VP35mut) known to have a diminished IFN antagonist activity in primate cells [25]. Our goal was to determine if (1) ERB cells could mount an effective antiviral gene program in response to a MARV infection, in either the presence or absence of immunomodulatory mechanisms by VP35, and (2) if so, how this profile might contrast with those reported previously for primate cell infections. We assessed differentially expressed genes (DEGs) using two transcriptomic platforms—RNA-seq and NanoString nCounter—the latter for which we developed a custom CodeSet of 221 ERB gene probes. Strikingly, analyses with both platforms revealed that, in our ERB cell line, the MARV infection almost completely blocked the immune gene stimulation, whereas MARV VP35mut led to the upregulation of many response genes, in keeping with the previously-described effects for primate cells. Interestingly, we also observed a marked lack of MARV-mediated *IFN* gene expression, induction of putative paralogs of novel, unannotated immune genes, and an enhanced basal expression of critical antiviral genes. Our findings suggest that, at least for this cell line and contrary to the leading hypothesis, the antiviral gene upregulation may not account for the difference in virulence between MARV-infected ERBs and primates.

## 2. Materials and Methods

### 2.1. Cell Culture

*Rousettus aegyptiacus*-derived immortalized kidney cell line RoNi 7.1 (a kind gift of Marcel Müller, University of Marburg) was cultured in Dulbecco’s Modified Eagle Medium (Gibco, Waltham, MA, USA), supplemented with 10% fetal bovine serum (HyClone, Logan, UT, USA) and antibiotics (Gibco). Cells were maintained at 37 °C with 5% CO_2_.

### 2.2. Virus Infection

All filoviruses were handled at a biosafety level 4 (BSL4) containment using the appropriate protocols and practices. RoNi cells were seeded at 5 × 10^4^ cells per well in 24-well plates. In triplicate, cells were mock-infected the next day using the media alone or infected at a multiplicity of infection (MOI) of 1 by the recombinant wild-type Marburg virus *R. aegyptiacus*-rec/UGA/2007/371Bat2007 (MARV WT) or the Marburg virus *R. aegyptiacus*-rec/UGA/2007/371Bat2007-VP35 R301A (MARV VP35mut), which has a mutation in VP35 (R301A) that abrogates its IFN antagonist activity. Infection by the Sendai virus (SeV) Cantell strain (Charles River Laboratories, Stone Ridge, NY, USA) was used at an equivalent amount as a positive control for antiviral responses. The media was refreshed after 1 h of adsorption and the plates were incubated at 37 °C until harvested.

### 2.3. RNA Isolation

Infected RoNi cells were harvested at 3, 8, and 24 h post-infection using 1× MagMAX RNA Lysis/Binding Solution Concentrate (Thermo Fisher Scientific, Waltham, MA, USA), after which the inactivated samples were removed from the biocontainment. The RNA was extracted by magnetic bead purification with the TURBO DNase treatment using the MagMAX-96 Total RNA Isolation Kit run in a MagMAX Express 96 Magnetic Particle Processor (Thermo Fisher Scientific). RNA purity and concentration was assessed by NanoDrop (Thermo Fisher Scientific) and stored at −80 °C.

### 2.4. Design of the ERB NanoString nCounter CodeSet

A custom NanoString nCounter gene expression CodeSet targeting 221 ERB-specific and 19 MARV- or SeV-specific genes was designed under contract by NanoString Technologies using the Raegyp2.0 genome assembly (GCF_001466805.2) and the NCBI *R. aegyptiacus* Annotation Release 100. Whenever possible, the probe designs were biased towards CDS and maximum transcript variant coverage. The selected ERB genes were focused on those implicated in key innate immune response pathways and common signaling pathways described in other mammals (Supplementary File S5). Genes preferentially expressed in peripheral blood mononuclear cells were avoided given the kidney-based origin of our RoNi cells. Additional probes were designed targeting MARV (GenBank FJ750958.1) and SeV Cantell clone cCdi (GenBank AB855654.1) transcripts to monitor viral gene expression and replication.

### 2.5. nCounter Hybridization and Data Collection

Hybridization reactions were performed in sets of 12 samples per run according to the manufacturer’s instructions. Briefly, hybridization buffer and RNase-free water were added to the ERB-specific Reporter CodeSet reagent, and this master mix was aliquoted into PCR reaction tubes. RoNi RNA was then added to each tube, followed by a Capture ProbeSet reagent. Samples were mixed, briefly spun down and incubated for 24 h at 65 °C in a T100 thermal cycler (Bio-Rad, Hercules, CA, USA). Each set was then stored at 4 °C until use, or loaded immediately onto an nCounter cartridge, which was run in an nCounter SPRINT Profiler for data collection.

### 2.6. nCounter Analysis

nCounter data were processed using nSolver 4.0 software (NanoString, Seattle, WA, USA) as follows. After quality control checks on individual RCC files, raw counts across samples were normalized to the geometric mean counts of synthetic DNA positive controls included in the hybridization reactions to mitigate platform-associated sources of variation. No background subtraction or thresholding was performed at this stage. Reference genes were selected using the geNorm algorithm within the nCounter Advanced Analysis (nCAA) module (version 2.0.115, NanoString) [26]. It identified the top five most stable genes in the sample set (ERCC3, POLR2A, G6PD, SDHA, and HDAC3). For each sample, normalization was performed by dividing counts for each gene by the geometric mean of the five reference genes. nCAA was used to calculate the differential gene expression (DGE) in infected RoNi cells relative to uninfected cells at the same time point (3, 8, or 24 h). The threshold for DGE was at least +/−1.0 log_2_ fold-change (FC) value and a Benjamini-Yekutieli-adjusted *p*-value < 0.05.

### 2.7. RNA-Seq Data Collection

RNA was evaluated for quality on the Agilent 2200 TapeStation. The libraries were generated on the Sciclone G3 Liquid Handling Robot (PerkinElmer, Waltham, MA, USA) using the TruSeq Stranded Total RNA Library Prep Kit (Illumina, San Diego, CA, USA). The library quality was assessed on the TapeStation and qPCR-quantified using the KAPA Complete Universal qPCR Kit (Kapa Biosystems, Wilmington, MA, USA) for Illumina. The libraries were diluted to 12 pM, cluster generation was performed on the Illumina cBot, and sequencing was run on the HiSeq 2500 using the paired-end 2 × 125 bp, dual-index format.

### 2.8. RNA-Seq Analysis

The read quality was verified by FastQC. Raw reads were trimmed on the edges for low quality reads/adapters and filtered using Trimmomatic V0.33 [27]. Paired reads were then aligned to the *R. aegyptiacus* transcriptome derived from the Raegyp2.0 genome assembly (GCF_001466805.2) and supplemented with manually-annotated Type I *IFN* genes and *NKG2* genes [22] with kallisto v0.43.0 [28]. A total of 100 bootstraps and default alignment parameters were used. Kallisto alignments were read into R v3.4.3. Data normalization was performed with sleuth [29]. DGE was performed with sleuth using the Wald test in a pairwise comparison. The threshold for DGE was at least +/−1.0 log_2_ FC with a False Discovery Rate (FDR)-corrected *p*-value (*q*-value) < 0.05. The processed RNA-seq reads were also aligned to MARV and SeV protein coding sequences (strains listed above) to obtain viral gene TPM values for the relative comparison to nCounter-derived viral gene counts. Fastqs and aligned files are found under GEO accession GSE117367.

### 2.9. Pathway Analysis

Ingenuity Pathway Analysis (IPA, Qiagen Bioinformatics, Hilden, Germany) was used to determine the significantly enriched pathways and upstream regulators for each infection condition.

### 2.10. Data Visualization

Data were plotted using the GraphPad Prism (v7.0) software (GraphPad Prism Software, Inc., La Jolla, CA, USA) and R v3.4.3 in RStudio v1.0.143.

### 2.11. GTEx Data Analysis

The public Genotype-Tissue Expression database [30] was used to determine transcript expression levels of individual genes and isoforms of two human-source cell types, kidney-cortex (45 datasets), and transformed fibroblasts (343 datasets). Outliers were removed from datasets if >2.5 standard deviations (SDs) from the mean. Mean and SD were then calculated for each gene and cell type.

## 3. Results

We conducted a transcriptomic analysis of an ERB kidney-derived cell line (RoNi) infected with a recombinant MARV derived from a wild-caught ERB isolate [24]. RoNi cells were mock-infected or infected at an MOI of 1 by either a wild-type (WT) MARV or a mutant MARV containing an amino acid change in the VP35 IFN inhibitory domain (IID) known to abrogate its ability to antagonize IFN induction in primate cells (MARV VP35mut) [25]. In parallel, RoNi cells were also infected with the Sendai virus (SeV) as a positive control for a robust antiviral response. Total RNA was collected at 3, 8, or 24 h post infection (hpi) and then analyzed using RNA-seq and NanoString nCounter transcriptomic platforms to simultaneously quantitate the host immune gene and viral gene expression. The relative abundance of the individual viral mRNAs is shown using the transcripts per million (TPM) metric for the RNA-seq and normalized probe counts for nCounter (Figure S1).

### 3.1. Differential Gene Expression for the nCounter CodeSet Strongly Correlates to RNA-Seq

**Figure 1:**
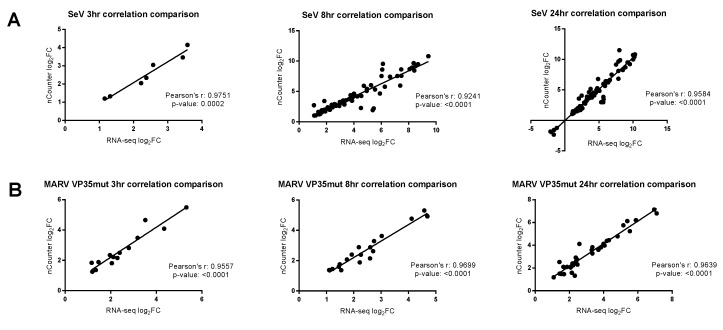
The correlation comparison between nCounter and RNA-seq. DEGs (differentially expressed genes) common to both the transcriptomic platform datasets were graphed against each other. RNA-seq log2FC (fold change) are shown on the *x*-axis and nCounter log2FC are shown on the *y*-axis. Pearson’s correlation analysis was performed to determine the relationships between datasets. The line represents the regression analysis. MARV WT correlation comparison is not shown because the two platforms shared no more than one DEG in common. (**A**) SeV comparison is shown for all three time points. (**B**) MARV VP35mut comparison is shown for all three time points.

**Figure 2:**
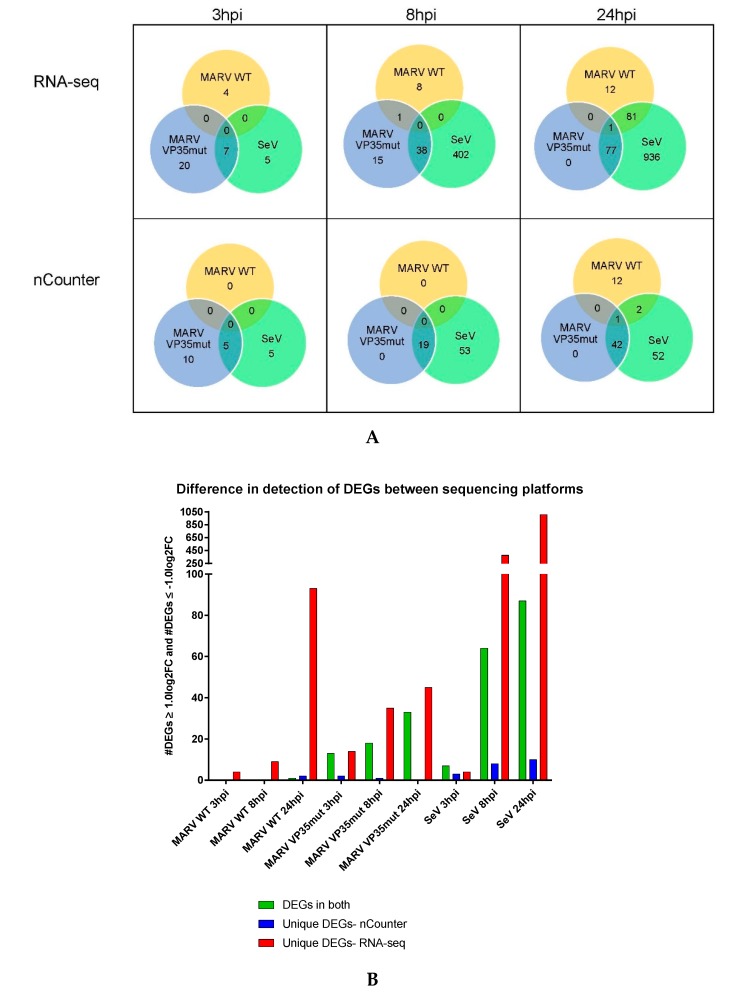
The shared DEGs between viruses within platforms and between platforms at each time point. (**A**) Numbers found in the center represent the total DEGs shared between all three viruses. The intersection of the green and yellow numbers represent the genes shared between SeV and MARV WT. The intersection of the blue and yellow numbers represent the genes shared between SeV and MARV WT. (**B**) The total number of DEGs in common for each transcriptomic platform are shown in green. The DEGs only found in the nCounter dataset are shown in blue. The DEGs only found in the RNA-seq dataset are shown in red.

To apply the faster, less laborious nCounter technology to the study of ERB immunobiology, we developed custom nucleotide probes targeting 240 host response, viral, and reference transcripts (CodeSet), and then validated their functionality against the gold standard RNA-seq method. These two techniques have been shown to correlate well in other human, NHP, and rodent studies. A differential host gene expression (DGE) analysis was performed on the two platform datasets in a pairwise manner between each virus infection condition versus control replicates. First, host DEGs identified by both platforms were plotted against each other to determine the data correlation. Analysis of each condition and time point resulted in Pearson’s r values above 0.92, indicating a strong correlation (Figure 1). We exempted MARV WT from this analysis because surprisingly only one gene at one time point (24 hpi) was shared between platforms due to an unexpected lack of RoNi antiviral gene induction described further below. Next, we compared the total number of DEGs obtained using either or both platforms to assess sensitivity differences and CodeSet limitations. It was anticipated that RNA-seq would identify far more overall DEGs, given the finite amount of gene probes in our ERB CodeSet (Figure 2, Supplementary Files S1 and S3). Regardless, most DEGs detected by nCounter overlapped with those found by RNA-seq, indicating that nCounter performs comparably to RNA-seq for any genes represented in a CodeSet (Figure 2). However, a limited number of DEGs were identified solely by nCounter, perhaps a consequence of the differing biochemistries with which each platform records the transcripts or how each defines the DEGs (Figure 2B, Supplementary File S5). Finally, also as anticipated, both platforms showed that SeV positive control infection induced the most DEGs of the three viruses. The majority of DEGs obtained for either MARV were found among these SeV DEGs (Figure 2A, Supplementary Files S1–S4).

### 3.2. MARV WT Does Not Induce a Canonical Antiviral Response Gene Program in RoNi Cells

**Figure 3:**
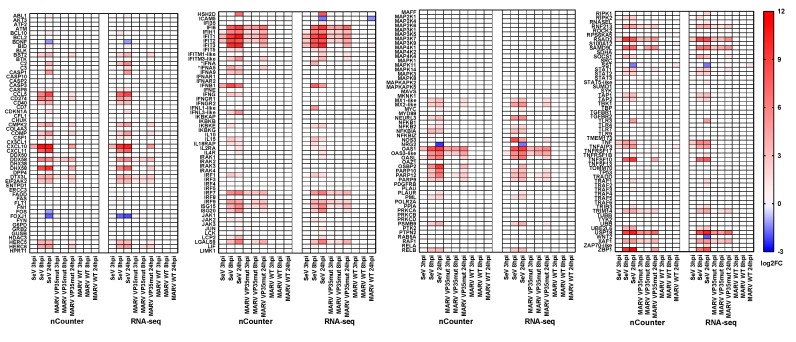
The nCounter codeset matched with DEGs from RNA-seq. A total of 221 ERB genes were chosen for the nCounter codeset, most of them involved in the antiviral responses. The scale is log2FC. *IFNA denotes the combined expression of the *IFNA* subtypes in the RNA-seq. No individual *IFNA* was significantly differentially regulated. *LTK and *IFI44* probes were developed using the *P. alecto* genome; these transcripts are not present in the transcriptome used for RNA-seq alignment. Genes described as “-like” are not officially annotated and the locus tags correspond as follows: *MX1*-like: *LOC107507192*; *MX2*-like: *LOC107507191*; *STAT5*-like: *LOC107513821*; *ZAP70*-like: *LOC107509971*; *IFNL1*-like: *LOC107521777*; *IFNL3*-like: *LOC107520938*; *IFITM1*-like: *LOC107506511*; *IFITM3*-like: *LOC107506941*; *OAS3*-like: *LOC107513228*.

Whereas SeV induced strong, broad immune gene expression as expected, RoNi cell infection by MARV WT led to drastically fewer DEGs at all time points (Figure 3, Supplementary Files S1 and S3). Only three DEGs were detected by nCounter at 24 hpi, *TNFAIP3*, *S100A12*, and *SST* (the only DEG detected by both platforms). Two additional CodeSet genes, *IFIT3* and *ICAM5*, were respectively up- and downregulated only in the RNA-seq dataset. Meanwhile, the more expansive analysis by RNA-seq revealed minimal MARV WT DEGs at early time points (only four at 3 hpi and nine at 8 hpi), but an increase to 94 DEGs at 24 hpi (12 unique, 81 shared with SeV and one common to all viruses) (Figure 2B, Supplementary File S4). However, most of these DEGs were not antiviral response-related, and many shared by SeV and MARV WT at 24 hpi were downregulated, potentially indicating a suppression by both viruses (Supplementary File S4).

To determine the biological significance of DEGs obtained by each platform, we used the Ingenuity Pathway Analysis (IPA), which ranked enriched canonical pathways and predicted upstream regulators. IPA gives an activation score (positive or negative z-score) that indicates whether a pathway is up- or down-regulated or an upstream regulator is predicted to be activated or deactivated, respectively. SeV at all three time points resulted in a strong upregulation of canonical innate immune pathways, and the top 20 predicted upstream regulators were consistent with those expected for a robust immune response activator (Figures S2 and S3A). However, no relevant antiviral signaling-related pathways were identified for any MARV WT DEGs. Together, our data suggest that MARV WT infection of RoNi cells dramatically suppresses antiviral response gene activation, similar to what has been previously reported for primate cells.

### 3.3. IFN Antagonism-Deficient MARV Infection Shows Robust Upregulation of Innate Immune Response Genes

Given the lack of immune gene upregulation in MARV WT-infected RoNi cells, we evaluated responses to MARV VP35mut infection. Consistent with expectations for the VP35 IID mutation, the analysis of MARV VP35mut showed a comparatively strong induction of many ERB antiviral genes at all time points, including those for IFN regulatory factor (IRF) activation and IFN signaling (Figure 3, Supplementary Files S1 and S3). Several key immune gene players were upregulated similarly to SeV control, such as *IFIT1*, *IFIH1*/*MDA5*, *DDX58*/*RIG-I*, *IRF7*, and *OAS1* (Figure 3). However, the MARV VP35mut induction profile was nevertheless distinct from SeV as the mutant virus showed a relatively reduced expression in a more limited number of genes (Figure 2 and Figure 3). Further, the kinetics of upregulation was delayed compared to SeV infection, with most DEGs only appearing at 24 hpi. Conversely, some genes were induced earlier by MARV VP35mut, including *RIG-I*, *IRF7*, *ISG15*, *IFI6*, and *UBA7*. MARV VP35mut also did not promote the downregulation of genes as observed for SeV and MARV WT.

**Figure 4:**
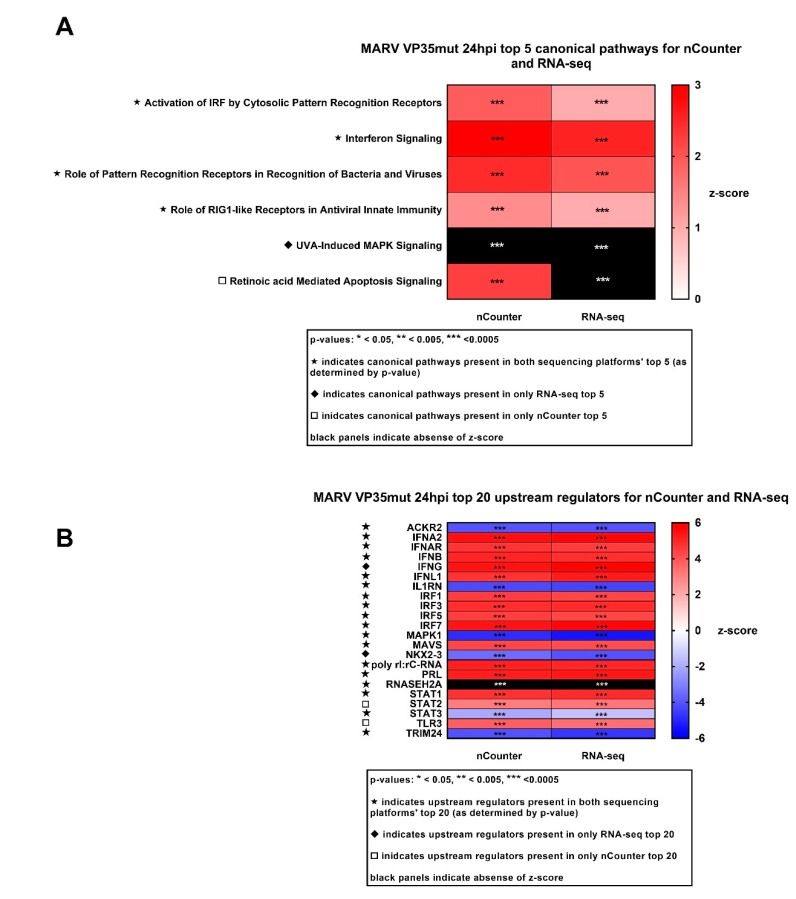
The top five canonical pathway and top 20 upstream regulators comparison between nCounter and RNA-seq for MARVmut. (**A**) The top 5 canonical pathways ranked by *p*-value. The key indicates which pathways are present in both platforms and which are present in only one. The z-score indicates an activation score calculated by IPA (Ingenuity Pathway Analysis) for each pathway. Positive values indicate that the genes in the dataset are behaving in a way that indicates positive regulation. Negative values indicate that the genes in the dataset are behaving in a way that indicates negative regulation. Black panels indicate canonical pathways that have a significant *p*-value but lack a z-score. (**B**) The top 20 upstream regulators ranked by *p*-value. The key indicates which pathways are present in both platforms and which are present in only one. The z-score indicates an activation score calculated by IPA for each pathway. Positive values indicate that the genes in the dataset are behaving in a way that indicates positive regulation. Negative values indicate that the genes in the dataset are behaving in a way that indicates negative regulation.

The IPA of the MARV VP35mut infection revealed top canonical pathways that included the “Activation of IRF by Cytosolic Pattern Recognition Receptors” and “Interferon Signaling” (Figure 4A, Figure S2B). The top four pathways at 24 hpi were identified by both platforms, and three pathways were shared by SeV control. IPA-predicted upstream regulators were also similar between platforms (Figure 4B). The top ten were dominated by genes from all three IFN types and by multiple IRFs, as expected for a virus with diminished VP35 IFN antagonist activity. Additionally present were ACKR2, a chemokine receptor for CCL ligands such as CCL5/RANTES, and IL1RN, an inhibitor of IL1, which is a proinflammatory cytokine produced by activated macrophages. Both predicted regulators had negative z-scores. Positive predicted regulators included canonical IFN induction players such as MAVS, TLR3, and IFN signaling players STAT1 and STAT2; additional relevant negative predicted regulators included MAPK1, STAT3, and TRIM24, the last of which plays an inhibitory role on STAT signaling. At early time points, most of the top predicted upstream regulators for MARV VP35mut were the same, and in a similar rank order, although IL1RN became a stronger hit over time (Figure 4B, Figure S3B). Conversely, RIG-I, among the earliest and most critical regulators of IFN induction, was the second highest-scoring prediction at 3 hpi and 8 hpi, but disappeared from the top 20 by 24 hpi, in keeping with its role as an antiviral response pathway initiator (Figure 4B, Figure S3B).

Another pathway that showed a notable upregulation of key genes and regulators was ISGylation, a distinct subset of the antiviral response in which ISG15, an ubiquitin-like modifier, is expressed and activated by proteolytic cleavage and linked to a variety of antiviral targets via an E1-E3 ligase chain (Figure S4A). Canonical pathway genes such as *RIG-I*, *IFIH1*, *EIF2AK2*/*PKR*, *MX1*, and *STAT1* were induced by SeV (Figure S4B). MARV VP35mut induced most of the same targets by 24 hpi, with the exceptions of *TRIM25* and *UBE2L6* (an E2 ligase gene). The expression of certain genes by MARV VP35mut earlier than SeV extended to several ISGylation regulators, including *ISG15* itself, *RIG-I*, *EIF2AK2*/*PKR*, *UBA7* (an E1 ligase), and *USP18* (the ISG15 processing peptidase).

Taken together, our findings suggest that the IID mutation of VP35 abrogates much of the MARV immune response antagonism in infected RoNi cells, as previously observed for primate cells [25]. Further, the mutation allows for a robust upregulation of antiviral genes, inclusive of key members of the canonical RIG-I, JAK-STAT, and ISG15 pathways, but with a more muted, targeted response than the broader transcription induced by the SeV control.

**Figure 5:**
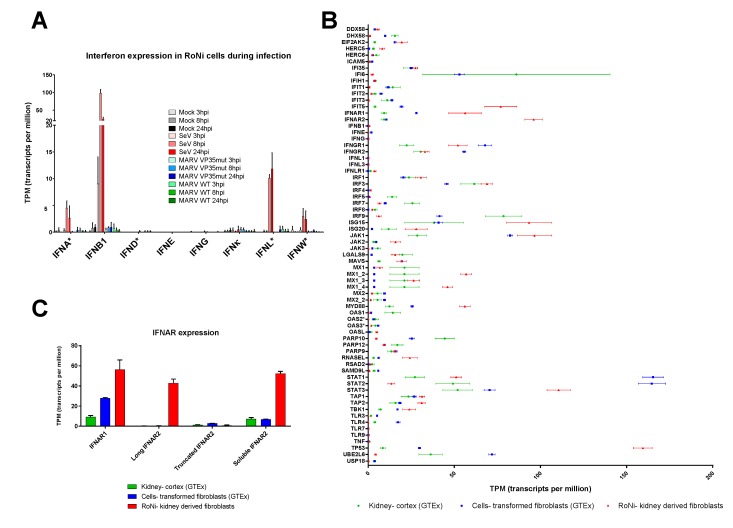
The *IFN* gene induction and baseline expression of innate immune genes. (**A**) The *IFN* gene induction in RoNi cells during infection. Transcripts per million (TPM) were graphed for each time point and infection condition. Error bars represent SD of three biological replicates. IFNs labeled with an * indicated aggregated expression of all subtypes. This includes the 12 *IFNA* subtypes, 22 *IFNW* subtypes, and 9 *IFND* subtypes. *IFNL* has not been annotated in the rousette genome, so this value represents the aggregation of LOC107520937, LOC107521776, LOC107520939, LOC107520938, and LOC107521777, all termed to be “*IFNL*-like” genes. (**B**) Samples include RoNi Mock cells (9 replicates) compared to transformed fibroblasts (343 replicates) and kidney-cortex cells (45 replicates) from the GTEx database. Outliers in the datasets were removed if the value fell beyond 2.5 SDs from the mean. Points represent the mean and the error bars correspond to SD. (**C**) Isoform expression of *IFNAR* variants is shown. Same datasets as in (**B**). Isoforms falling into the categories of long, truncated, and soluble were aggregated and plotted. Starred genes correspond as follows: *OAS2: LOC107501264; OAS3: LOC107513228; MX1: LOC107498547; MX1_2: LOC107504926; MX1_3: LOC107507190; MX1_4: LOC107507192; MX2: LOC107507191; MX2_2: LOC107507193; IFNL1: LOC107521777; IFNL3: LOC107520938.*

Finally, while both platforms detected DEGs for SeV and MARV VP35mut that are known in primates to be regulated by all IFN types, and even a select few modestly altered for MARV WT, we discovered that, unexpectedly, the MARV virus did not induce any *IFN* gene at any time point, with the exception of *IFNA5* at 24 hpi for MARV VP35mut (as detected by nCounter, with probe designed for *IFNA5* that likely binds to several *IFNA* subtypes) (Figure 3 and Figure 5A). This striking lack of IFN expression appeared MARV-specific, as SeV induced IFNs of all types, including *IFNB*, *IFNG*, and *IFNL1*-like and *IFNL3*-like genes, at multiple time points. In this analysis, the MARV viruses failed to induce *IFNW*, which was induced by SeV, albeit at low levels, at 24 hpi, along with every other class of IFN (Figure 5A). Meanwhile, *IFND*, *IFNK*, and *IFN*G showed a minute basal expression in RoNi cells, and *IFNE* expression was completely absent. These data are even more intriguing in light of recent description by our lab of evolutionarily-expanded *IFN* gene loci in ERBs, including those for *IFNA* and *IFNW* classes [22].

### 3.4. Expanded Immune Gene Repertoire Is Upregulated in RoNi Cells

To further characterize the RoNi cell response to a MARV infection, we examined other factors that may contribute to previously hypothesized, host species-specific viral tolerance [22]. As the ERB genome was only recently mapped, many genes remain unannotated due to a lack of one-to-one orthologs or to being part of an expanded gene locus. Many of these unannotated or expanded genes were found to be DEGs after the analysis of our RNA-seq dataset (Figure S5, Supplementary File S3). These locus tag DEGs were categorized by the NCBI eukaryotic genome annotation pipeline as *Ig lambda*-, *HLA class I/II*- or *IFNL3*-like genes, as well as several antiviral response-related or uncharacterized genes. Many of the locus tag DEGs appear to be paralogs of expanded immune genes, including canonical antiviral genes such as *MX1*, *OAS3*, *ISG20*, *IRF4*, and *IFIT1*. Interestingly, several genes, like *IFNL*, *MX2*, and *PARP14*, had multiple distinct copies detected. These putative, noncanonical immune gene paralogs have not been functionally described, but could contribute to the ERB tolerance to viral infection.

### 3.5. Basal Expression of Many Antiviral Genes Is Higher in Uninfected RoNi Cells Compared to Human Cells

Along with the potential expansion of critical response genes, we found that the baseline expression of many others was naturally elevated in uninfected RoNi cells compared to analogous human cell types (Figure 5B). This was assessed by comparing expression data from the GTEx project database of kidney-cortex cells (45 total datasets) and a transformed fibroblast cell line (343 total datasets) to our mock RoNi dataset. Several RoNi genes with significantly higher basal levels were response regulators or signaling factors, including *ISG15*, *ISG20*, *RNASEL*, *IFIT5*, *IRF3*, and *IRF1* (Figure 5B). Other potentially important genes, such as *TP53* and *STAT3*, were also elevated in RoNi cells. Contrary to previous reports for *P. alecto* cells, *IFNA* genes were not constitutively expressed in RoNi cells [20]. Instead, we found twice the expression of Type I IFN receptor *IFNAR1*, and dramatically higher levels of full-length (long) *IFNAR2* as well as its soluble isoform, the most abundant *IFNAR2* transcript. In contrast, these *IFNAR2* variants showed little to no expression in human cells (Figure 5B,C).

## 4. Discussion

Our study sought to determine the ERB antiviral state in vitro upon a MARV infection. We used an ERB kidney-derived cell line (RoNi/7.1 cells) as a surrogate for bats and previously published, recombinant MARV viruses derived from a wild-caught ERB isolate [24] to avoid the possible adaptation to primates. We find that MARV WT infections strongly suppresses antiviral responses in RoNi cells, while infection with IFN antagonism-deficient MARV VP35mut results in a significant antiviral gene expression. The results highlight the immunosuppressive role of MARV VP35 and are equivalent to those observed previously in primate cells. Strikingly, the antiviral gene upregulation in MARV VP35mut-infected RoNi cells occurs amid a near-absent upstream *IFN* induction. We also report the differential expression for several novel putative antiviral gene paralogs as well as uncharacterized genes. Finally, compared to similar human cell types, we observed an enhanced basal expression of several genes that may be involved in ERB antiviral activities.

While RNA-seq remains the gold standard transcriptomic platform, encompassing as many genes as discovered, its use is hindered by a specialized lengthy analysis. An alternative is the NanoString nCounter, which is advantageous due to lower costs for smaller scale experiments, and simplified analysis, but is limited by gene capacity in a CodeSet. We designed the first known ERB CodeSet with 240 host response, viral, and reference genes and compared its performance to RNA-seq. Indeed, even with a CodeSet comprising only ~1% of the annotated ERB genome, we obtained a comprehensive picture of the in vitro antiviral gene program occurring in MARV-infected ERB cells. We conclude that nCounter, expandable up to 800 gene probes, is a promising first-line tool for any future ERB transcriptomic study where CodeSet capacity does not impede the desired analytical scope.

Given the opposing clinical phenotypes observed between MARV-infected ERBs and primates, we hypothesized that MARV-infected RoNi cells would show a much more robust antiviral response compared to the near-total suppression reported in primate cells. Instead, we discovered that primate cell-like antiviral gene suppression by MARV also occurs in RoNi cells. Our results were largely concordant with previous findings in MARV-infected ERB cells [18]. However, the authors of that study also found some key antiviral response genes apparently deregulated at certain time points, including *RIG-I*, multiple *STAT* genes, *HERC5*, and *MX1*, which we did not detect. Another ERB cell-based study by Kuzmin et al. had even more divergent results, as they detected a strong response gene induction by a human MARV isolate, including many critical antiviral genes such as *IFIT2*, *MX2*, and *IRF9* [17]. Further, when using the same bat isolate-derived MARV virus as in our study to infect RoNi/7 cells, they reported a 100-fold upregulation of *IFIT1* via qPCR. While the experimental design between their publication and ours appears similar, we do note that usage of a different bat cell line, techniques for the rescue and propagation of virus (for instance, unnecessarily freeze-thawing infected cells), and/or differences in how transcriptomic analyses and qPCR-based assays were performed could each be responsible for the described discrepancies.

We observed a difference in the viral gene expression kinetics of the two recombinant MARVs (Figure S1). Surprisingly, MARV VP35mut appeared to replicate earlier than MARV WT. As MARV VP35mut is known to lack VP35-mediated IFN antagonism and be attenuated for growth in vitro in primate cells [14,25], we did not expect a lower viral gene expression by the WT virus in RoNi cells. However, this lower MARV WT expression is unlikely to account for the observed absence of ERB responses, as MARV WT at 24 hpi shows a greater viral expression than does MARV VP35mut at 8 hpi, while still clearly showing a lack of response gene induction. Further, many MARV WT DEGs at 24 hpi that are not antiviral-related coincide with those found for SeV, which simultaneously maintained a stimulation of an active immune response. This indicates that RoNi transcriptomic profiles are due to the protein activity of each virus rather than due to differences in viral gene expression.

Our results from both the WT and VP35mut viruses indicate that MARV VP35 is fully capable of antagonizing antiviral gene induction in RoNi cells. This is not unprecedented, as both the Ebola and Lloviu virus VP35 proteins were recently found to strongly inhibit IFN induction in an *Epomops buettikoferi* African fruit bat cell line [31]. Although we assume that MARV VP35 immunomodulatory mechanisms in RoNi cells mirror those in primate cells (i.e., preventing RIG-I activity, inhibiting IRF3, and disrupting MAVS and IRF7 interactions), our current results observed in other innate immune sensing pathways [22] suggest that protein-based studies will be needed to confirm that these functions apply in ERBs [32,33,34,35,36,37].

Despite far greater MARV VP35mut-induced antiviral gene expression compared to MARV WT in RoNi cells, the overall response was distinctly lower and more specific than for SeV. This could be due to the suppression by other MARV proteins. MARV matrix protein VP40 is also a known IFN antagonist, blocking the JAK1 activation of downstream IFN signaling [14,16], and a putative MARV GP immunomodulatory domain has yet-unknown effects in ERBs [38]. As with VP35, efforts to define the roles and functions of these other viral proteins in infected ERBs will be insightful. Lastly, while the MARV VP35mut response phenotypes in RoNi cells were largely similar to those previously reported in primate cells, we did not expect the absence of *IFN* gene induction. It remains speculative as to how these responses might occur, but there is a precedent for noncanonical, IFN-independent antiviral gene upregulation. Such novel pathways could involve interactions with IRF1, IRF3, and IRF7, which can function in lieu of IFNs [39,40,41]. We must also recognize that the current “canonical” IPA is based on well-studied human responses and pathways, and may not reflect uncharacterized, potentially-unique ERB pathways, factors or interactions.

Among the canonical pathways upregulated by MARV VP35mut in RoNi cells, ISGylation was one of the most consistent and robust. ISGylation plays an important role in the human cell response to filovirus infections. ISG15 is an ubiquitin-like modifier, but unlike ubiquitination, it appears to disrupt activity or localization of targets rather than direct them for proteasome-mediated degradation [42]. It is implicated in EBOV restriction via interference with NEDD4-mediated ubiquitination of VP40, which impairs viral budding [43,44]. ISG15 may restrict MARV VP40 by an analogous mechanism. Inhibition of budding in RoNi cells could help explain the higher viral gene counts for MARV VP35mut, as this virus greatly induces ISG15, potentially confining more immature virus within cells. Regarding additional relevant pathways, RNASEL, an OAS-regulated enzyme that targets and degrades foreign dsRNA was found to be a key component of antiviral responses in IFN-treated *P. alecto* cells [45]. However, we saw only a marginal induction of *RNASEL* in SeV-infected RoNi cells at 24 hpi, even with strong *OAS* gene stimulation at all time points, and no upregulation by either MARV virus. Other, more highly-induced immune genes may play bigger roles than *RNASEL* in the ERB antiviral response. Interestingly, our data also generated ERB-specific response genes that have not been previously identified or annotated. These locus tag genes include putative paralogs for *OAS2*, *MX2*, *IFIT1* and, especially, *PARP14*, which had up to seven upregulated at once. The evolutionary expansion of these novel ERB genes and respective roles that any resultant protein products play in host defense or other processes will be a promising avenue for future molecular studies.

Finally, we noted an elevated basal expression of many response genes in uninfected RoNi cells, including *ISG20*, *IFNAR2*, and *STAT3*, compared to the collated data from related human cell types. For example, the resting *RNASEL* transcription was significantly higher than in the human dataset. We speculate that this gene and others could exist in a basally “induced” state in RoNi cells. This may explain why some genes, like *RNASEL*, showed a muted stimulation even during a robust response, as their promoters were already active. Another gene with enhanced basal expression was *ISG15*, which, in this case, might be at least partially IRF-dependent. In human cells, IRFs can weakly and transiently transactivate *ISG15* without IFN signaling [46,47]. However, in RoNi cells, higher IFNAR levels, especially soluble IFNAR2—a putative potentiator of quicker more sustained responses—could enable strong IFN-independent, IRF-mediated *ISG15* induction, allowing cells to engage a core aspect of the ISGylation pathway in advance of the infection [48]. Interestingly, the cell cycle regulator TP53 can also transactivate both *ISG15* and certain *IRF* genes [49,50]. We found *TP53* among the highest basally-expressed response genes in RoNi cells compared to human cells. Given these intriguing possibilities, we further speculate that this proposed basal induction, including those for major canonical antiviral regulators and receptors, could help prime RoNi cells for a rapid, more efficient host response coordination and infection control in an IFN-independent manner, perhaps an ancient driver of MARV adaptation via VP35. Nevertheless, as our study employed an immortalized cell line, we cannot rule out that higher basal transcription may be artifactual and RoNi cell specific.

In conclusion, our transcriptomic analyses of MARV-infected RoNi cells suggest it is unlikely that antiviral gene upregulation alone can explain ERB resistance to severe MARV disease. Our findings reveal that immune suppression is still a critical feature of MARV infection in ERB cells. Antiviral gene induction is presumably only one of a series of host defenses that MARV must overcome in order to remain viable in ERBs, as multiple determinants of disease resistance might exist. Various host gene activities, pathways, or processes could resist but still tolerate a MARV infection. ERBs may have noncanonical defense mechanisms, as suggested by our observation of IFN-independent signaling and the possible basal “induction” of critical response genes. In fact, we posit that a co-evolved interplay between MARV-mediated immune gene suppression and host-mediated noncanonical responses likely defines the ERB disease resistance by tempering both overactive MARV replication and host proinflammatory activation [22]. However, our observations may not be applicable to other bat species, as each could have evolved unique strategies to tolerate their set of species-specific pathogens. Admittedly, given the limitations inherent to using RoNi cells as an in vitro ERB model, it is impossible to capture all aspects of the antiviral response that might otherwise occur in the context of a whole animal (bat) infection. In primates, MARV inhibits antiviral responses in vitro but elicits an exacerbated inflammatory response in vivo, and the balance of both effects may contribute to the severe disease phenotype. Therefore, in vivo ERB infection studies will be vital to our continued exploration for immune response factors that dictate avirulence in the MARV reservoir host.

## Supplementary Materials

The following are available online at http://www.mdpi.com/1999-4915/10/11/607/s1, File S1: nCounter DEGs, Files S2: nCounter shared targets; File S3: RNA-seq DEGs; Files S4: RNA-seq shared targets; File S5: nCounter raw and normalized counts.
