# Supplementary material for: Transcriptomics Reveal Antiviral Gene Induction in the Egyptian Rousette Bat Is Antagonized In Vitro by Marburg Virus Infection
Source: Viruses. 2018 Nov 2;10(11):607. doi: 10.3390/v10110607 (PMC6266330; doi:10.3390/v10110607)
Supplement: Supplementary file 1 [file viruses-10-00607-s001.zip › viruses-375811-supplementary/Supplemental Files/viruses-375811-supplementary figures.pdf]

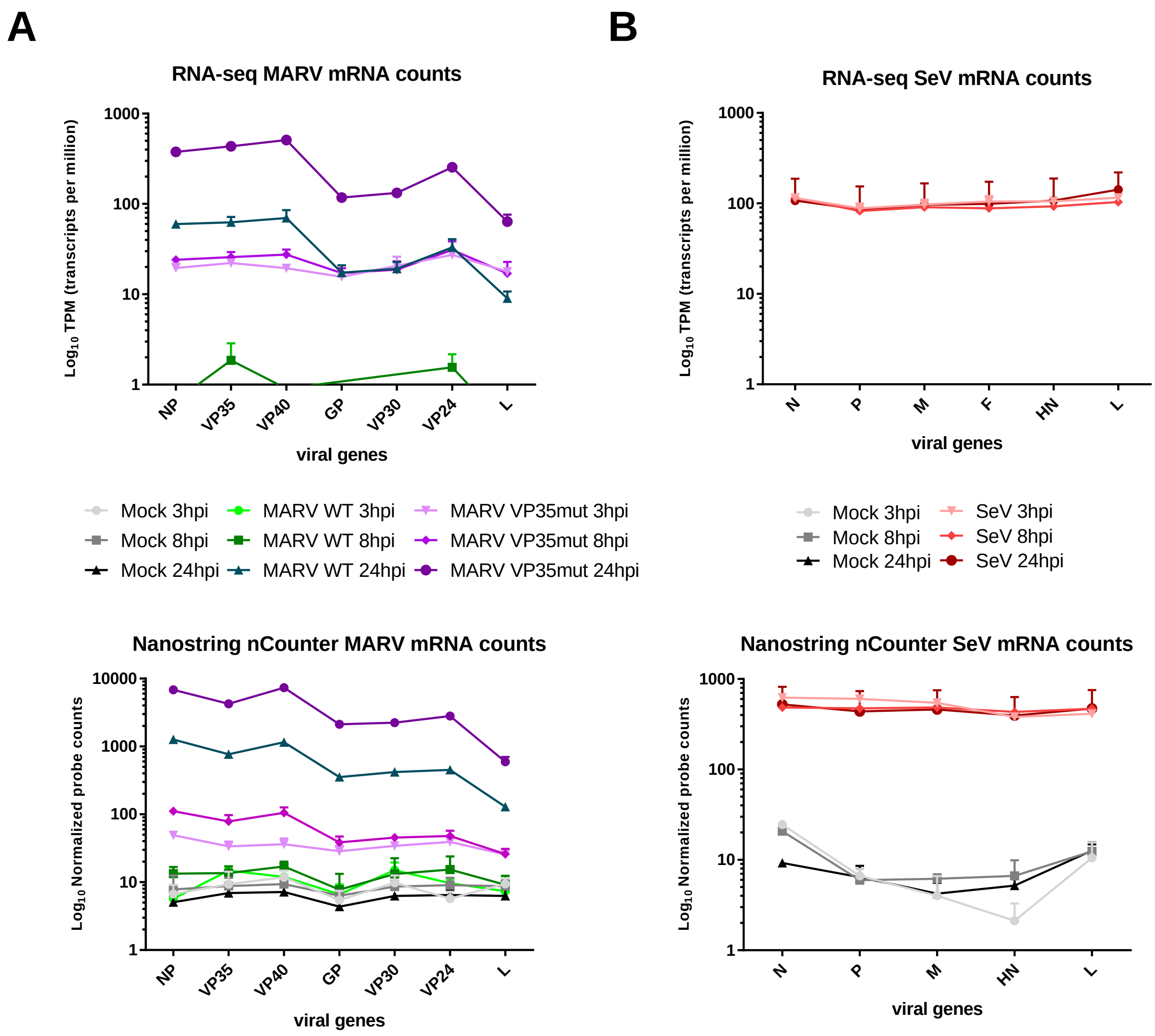

Figure S1. Viral gene expression for nCounter and RNA-seq platforms. RNA-seq reads quantified in $\log_{10}$ transcripts per million (TPM), a metric that accounts for total reads and gene length bias. Transcriptome alignment allows quantitation of viral genes and means plotted with error bars representing SD. nCounter reads quantified in $\log_{10}$ normalized probe counts and means plotted with error bars representing SD. **A.** MARV WT and MARV VP35mut counts shown. **B.** SeV counts shown.

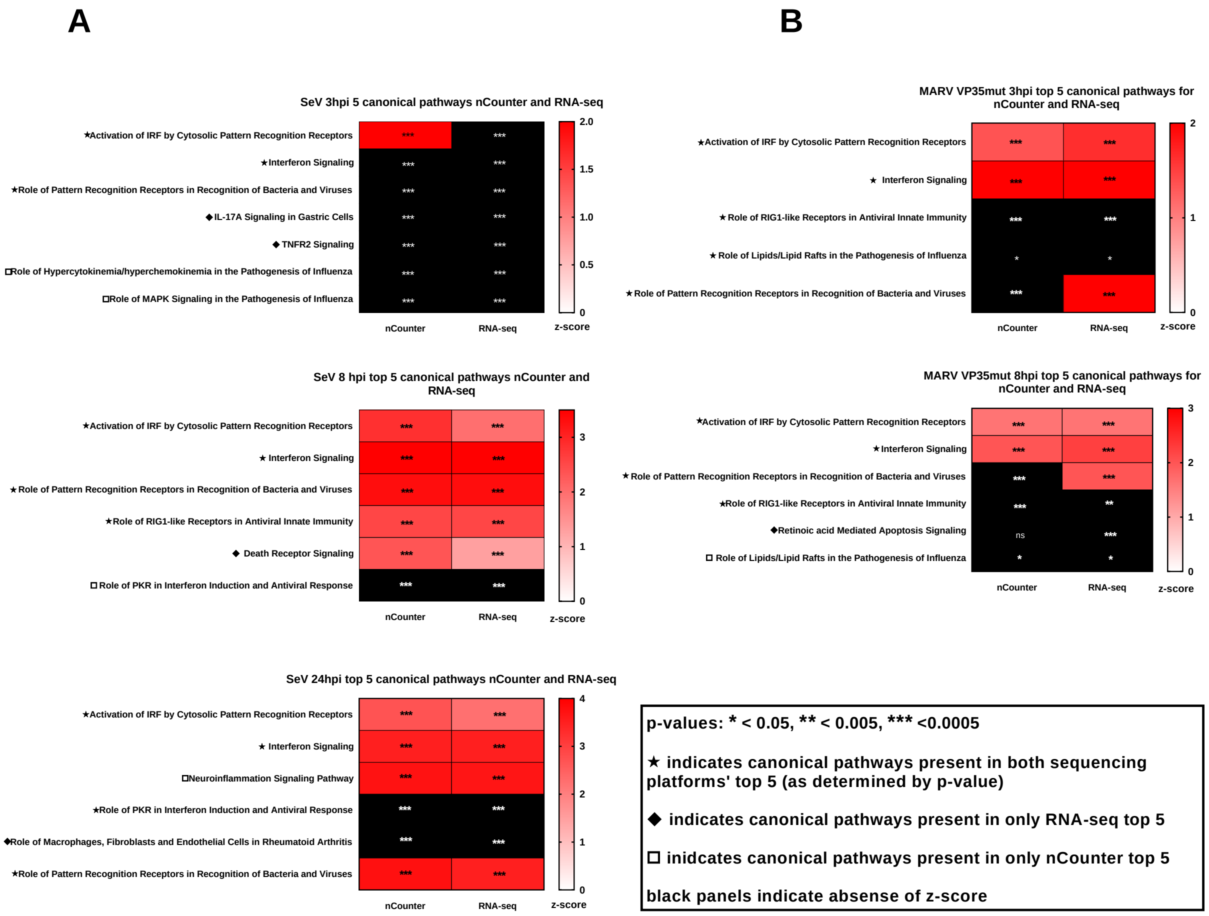

Figure S2. Top five canonical pathway comparison between nCounter and RNA-seq for MARV VP35mut and SeV. Top five pathways ranked by p-value and listed by alphabetical order. Key indicates which pathways are present in both platforms and which are present in only one. Z-score indicates an activation score calculated by IPA for each pathway. Positive indicates that the genes in the dataset are behaving in a way that indicates positive regulation. Negative indicates that the genes in the dataset are behaving in a way that indicates negative regulation. Black panels indicate canonical pathways that have a significant p-value but lack a z-score. **A.** SeV canonical pathways for 3 hpi, 8 hpi, and 24 hpi. **B.** MARV VP35 canonical pathways for 3 hpi and 8 hpi.

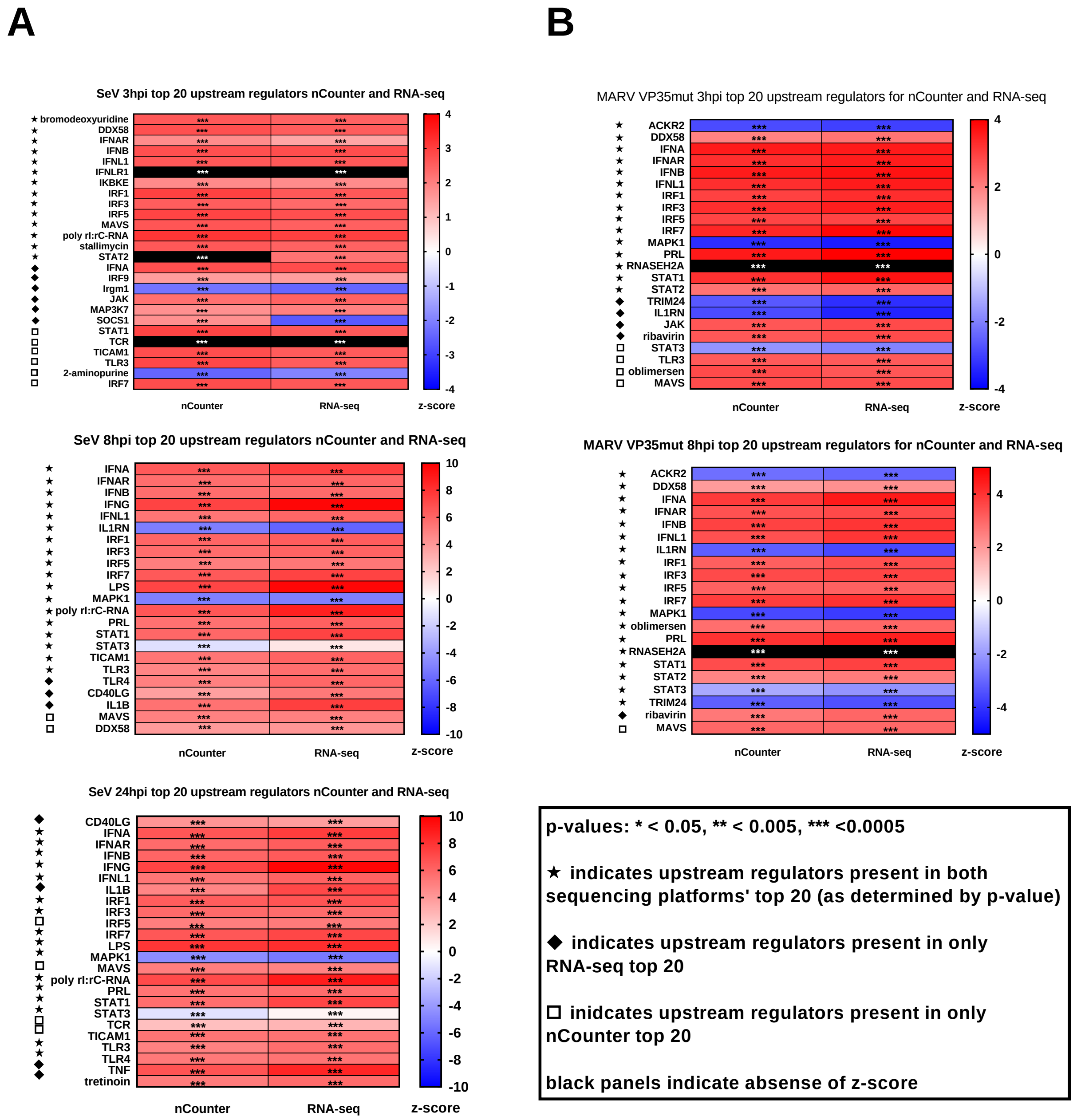

Figure S3. Top 20 upstream regulators ranked by p-value. Key indicates which pathways are present in both platforms and which are present in only one. Z-score indicates an activation score calculated by IPA for each pathway. Positive indicates that the genes in the dataset are behaving in a way that indicates positive regulation. Negative indicates that the genes in the dataset are behaving in a way that indicates negative regulation. **A.** SeV upstream regulators for 3 hpi, 8 hpi, and 24 hpi. **B.** MARV Vp35mut upstream regulators for 3 hpi and 8 hpi.

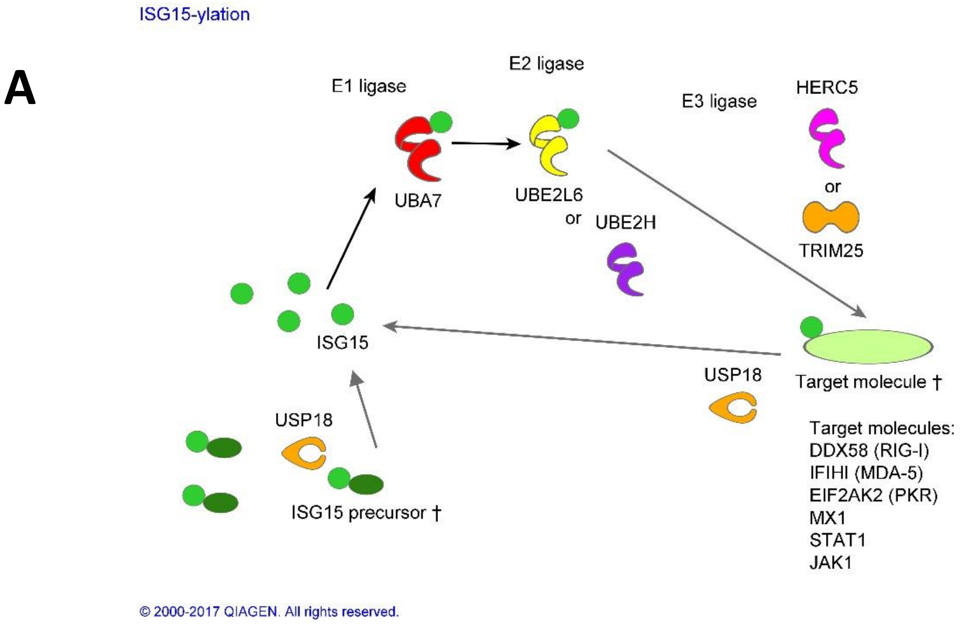

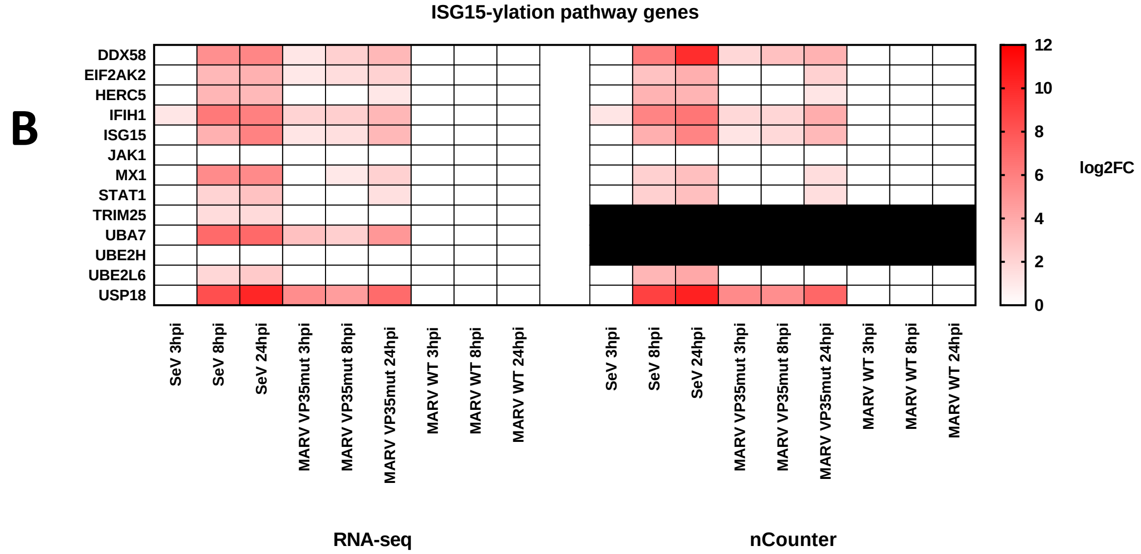

Figure S4. ISGylation pathway components and their expression during infection. A. A cartoon shows the mechanism of ISGylation during infection. ISG15 is cleaved from its precursor by USP18 to generate mature ISG15. ISG15 is activated by and linked to an E1 ligase, then transferred to an E2 ligase, and finally linked to a target molecule through an E3 ligase. **B**. Expression of relevant genes within the ISGylation pathway from the nCounter and RNA-seq datasets.

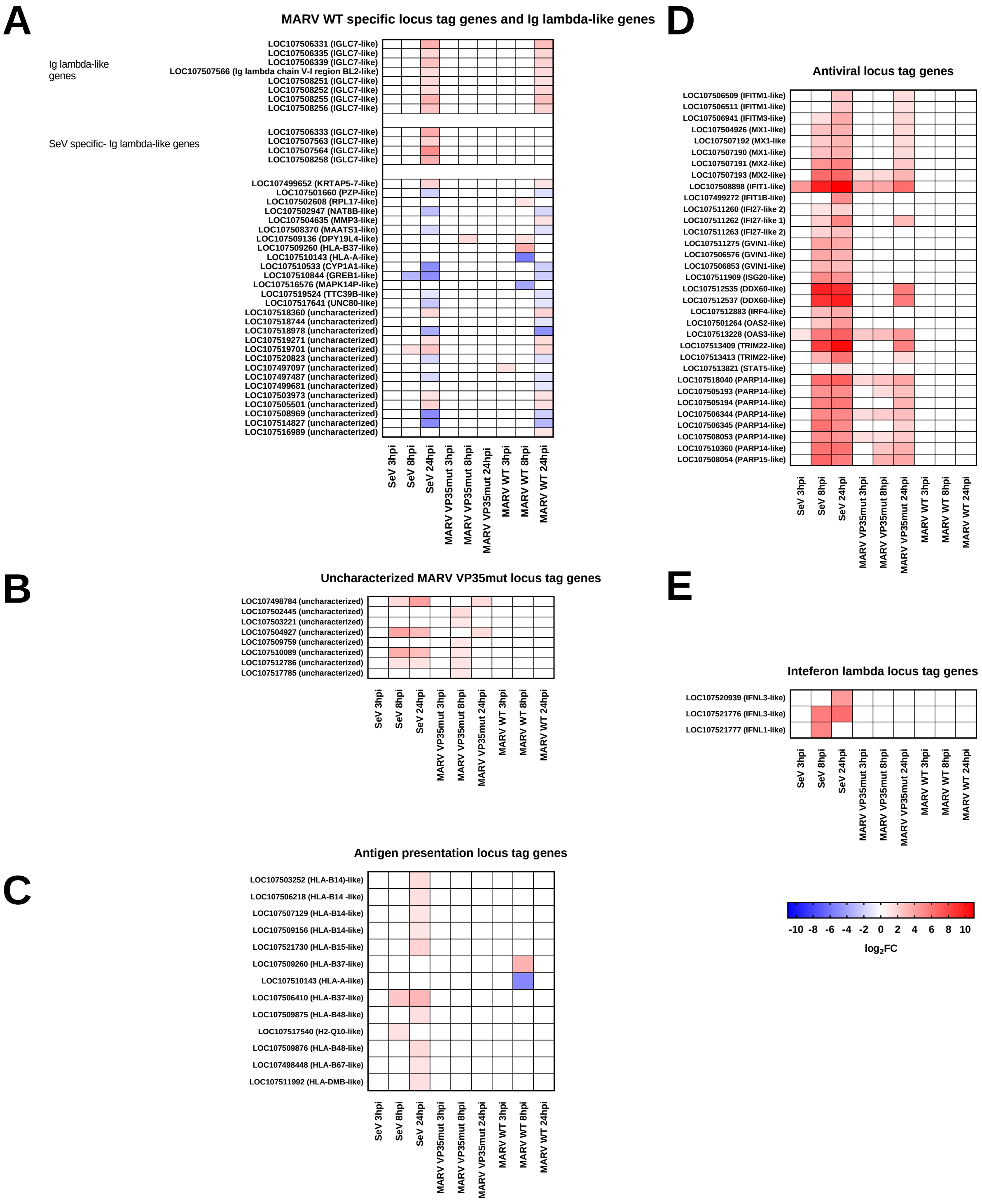

Figure S5. Differentially expressed locus tag genes during infection. A. Locus tag genes corresponding to Ig lambda-like genes and all locus tags found in MARV WT datasets. **B.** Locus tag genes corresponding to uncharacterized genes during MARVmut infection. **C.** Locus tag genes corresponding to antigen presentation-like genes. **D.** Locus tag genes corresponding to antiviral response-like genes. E. Locus tag genes corresponding to *IFNL*-like genes.
